# Development and validation of the Harm Concept Breadth Scale: Assessing individual differences in harm inflation

**DOI:** 10.1371/journal.pone.0237732

**Published:** 2020-08-18

**Authors:** Melanie J. McGrath, Nick Haslam

**Affiliations:** Melbourne School of Psychological Sciences, University of Melbourne, Parkville, Victoria, Australia; Aalborg University, DENMARK

## Abstract

Three studies (*N*s = 350, 301 & 341) examined the reliability, validity, and correlates of a new measure of harm inflation, the individual differences counterpart of ‘concept creep’. The Harm Concept Breadth Scale (HCBS) assesses variability in the expansiveness of concepts of harm (i.e., bullying, mental disorder, prejudice, trauma), such that these concepts refer to a wider range of phenomena among people scoring high on the scale. Study 1 developed 66 vignettes representing potential instances of the four concepts, selected optimal subsets of 10 vignettes for each concept, and demonstrated satisfactory internal consistency of the subscales. Study 2 demonstrated that the HCBS had excellent internal consistency, and established construct validity through associations with measures of moral foundations, justice sensitivity, general category inclusiveness, and political orientation. Study 3 employed participants from a different national background and further clarified the correlates of the HCBS via measures of empathy, moral expansiveness, and the Big Five personality traits. The findings indicate that concept breadth is a reliably measurable individual difference variable with weak to moderate associations with harm-based morality, prosocial concern, political liberalism, female gender, and negative emotionality. The HCBS is a valid psychometric instrument for examining the causes and implications of harm inflation.

## Introduction

Western societies appear to be increasingly preoccupied with harm. Although there is evidence that life in these societies is becoming safer [1], our cultures pay growing attention to abuse, bullying, harassment, mental health problems, prejudice, and trauma. The rising salience of harm has often been understood through two narratives. In one, often associated with the political left, our societies are becoming more aware of previously ignored or tolerated forms of harmful behavior and experience, and are taking steps to address them. In this view, our growing attention to harm represents a progressive expansion of care and moral concern for others. In the second narrative, often associated with the political right, the rising emphasis on harm reflects over-sensitivity, weakness, and vulnerability. In this view, the rising salience of harm is creating a generation of fragile and aggrieved victims. Understanding the psychology of these cultural trends and clarifying their implications is a priority for researchers.

One attempt to make sense of the rising salience of harm was made by Haslam [2] in his work on ‘concept creep’. Haslam argued that harm-related psychological concepts such as prejudice, bullying, trauma, and mental disorder, have gradually expanded the range of phenomena to which they refer in recent decades. This semantic inflation takes two forms. Horizontal creep occurs when an existing concept comes to be applied to qualitatively new phenomena (e.g. the concept of school yard bullying extended to the workplace or online environment), whereas vertical creep occurs when a concept broadens to encompass milder variants of the phenomenon in question (e.g., ‘trauma’ coming to include vicarious rather than personal experiences).

Although semantic expansion has been identified independently in numerous domains of psychology and psychiatry [3–6], Haslam [2] proposed a unifying explanation. Concept creep, he argued, is confined to harm-related concepts and reflects an increasing sensitivity to harm. Vertical creep enables increased sensitivity to harm by lowering the threshold for identifying harm (e.g., defining increasingly subtle acts as examples of prejudice) and horizontal creep does so by recognizing new forms of harm. Haslam’s claim that concept creep may be driven by a rising cultural concern with harm is supported by a recent examination of the relative frequency of harm-related words in the Google Books database. This analysis found that these words have risen steeply in salience since 1980 [7].

If understandings of harm-based psychological concepts are undergoing a dynamic process of expansion, a cross-section of individuals within the culture might demonstrate different rates of adoption of these shifting meanings. For example, older people may retain the narrower definitions of concepts that prevailed when they were raised. Alternatively, the inclusiveness of concept definitions may be influenced by life experiences, personality traits, or political beliefs. We refer to these individual differences in concept definitions as differences in “concept breadth”.

### Harm-related concept breadth

Harm-related concept breadth represents an individual’s tendency to hold expansive definitions of concepts concerned with psychological harm. As a construct linked to the phenomenon of concept creep it is distinct from related individual differences constructs such as harm avoidance [8], endorsement of the harm moral foundation [9], and empathic concern [10]. Its cognitive and linguistic focus on word meanings distinguishes it from harm avoidance and empathic concern’s focus on affective reactions to experiences of suffering, and from the moral values and judgments embedded in harm-based morality.

The present research attempts to develop and validate a measure of this new individual difference construct. Such a measure would enable research related to concept creep, clarifying its psychological foundations and implications by addressing predictors and consequences of cross-sectional individual differences. A way of assessing harm-related concept breadth would also be very timely in view of the political dimension of concept creep. Disagreements about what is harmful underpins much of the moral conflict between liberals and conservatives [11], and differences in harm-related concept breadth may thus contribute to ideological polarization. The capacity to identify variation in breadth and its antecedents may inform efforts to mitigate the detrimental effects of this polarization. A greater understanding of factors linked to increased sensitivity to concepts such as trauma and mental disorder might also prove valuable in identifying potential vulnerabilities to psychological harm. The series of studies reported here develop and validate a measure that can be used for these important purposes.

Earlier work investigating individual differences in breadth of harm-related concepts utilised a preliminary 24-item measure comprising six items each for bullying, prejudice, trauma and abuse [12]. Although suitable for initital exploration of the construct, this measure had several limitations. Items were not systematically constructed or selected, with a priori judgments made regarding what represents ‘narrow’ or ‘broad’ instances of a concept. The concept subscacles also did not include sufficient items to allow breadth in indivdual concepts to be measured reliably. The development and validation of the Harm Concept Breadth Scale addresses and remedies these limitations.

### The Harm Concept Breadth Scale

The Harm Concept Breadth Scale (HCBS) is intended to capture variation in an underlying construct that influences the expansiveness of a variety of harm-relevant psychological concepts. The scale comprises concept-specific subscales assessing the breadth of each concept, with the full scale assessing a generalized harm-related concept breadth factor. The concepts were selected from those identified in Haslam’s [2] concept creep theory paper, which presented case studies on six concepts: prejudice, trauma, mental disorder, bullying, addiction, and abuse. The latter two, abuse and addiction, were eliminated due to their potential overlap with the other concepts. The four selected concepts are not intended to be an exhaustive set of ‘creeping’ concepts, but to sample the broader domain of harm-related concepts. The HCBS utilises brief vignettes describing social situations that may or may not represent instances of each concept. The vignette format allows concept inclusion or exclusion judgments to be made about concrete examples rather than requiring participants to generate abstract concept definitions. The more examples a participant judges to be instances of harm-related concepts, the broader (i.e., more inclusive) those concepts are inferred to be.

We report here on the development and validation of the HCBS. Study 1 developed the scale from a vignette item pool and sought evidence of its reliability and factor structure. Study 2 further tested the reliability of the finalized scale and tested its structural and construct validity. This study also began the work of putting the HCBS into a nomological network by exploring potential predictors of its construct. Study 3 continued the construct and structural validation process with a demographically different sample. All studies were approved by the University of Melbourne’s Human Research Ethics Committee and written consent was provided by all participants.

## Study 1: Development of the Harm Concept Breadth Scale

The purpose of Study 1 was preliminary scale development of the HCBS, including refinement of an item pool, testing of internal consistency of subscales, and preliminary assessment of its latent structure, with the expectation that the four subscales load on a superordinate harm-related concept breadth factor.

### Method

#### Participants

350 participants comprising 194 men (55.4%), 154 women (44.0%) and two people identifying as non-binary (0.6%) were recruited from Amazon Mechanical Turk (MTurk). To be eligible for the study MTurk Workers were required to be residents of the United States and to have completed at least 1000 MTurk tasks, with an approval rate of ≥98%. Participant age ranged from 18 to 74 with a mean of 36.53 years (*SD* = 10.81). A majority (70.3%) of participants identified as White or Caucasian, 8.3% as African American, 7.7% as Hispanic or Latinx, and 7.7% as Asian. Other ethnicities accounted for the remaining 6.0%.

### Materials and procedure

#### Harm concept breadth item pool

A literature review focusing on definition and measurement was conducted for each of the four concepts to be included in the final scale (prejudice, bullying, mental disorder, and trauma). These reviews informed matrices capturing horizontal and vertical expansion of the concepts over time within the respective literatures. That variation was then sampled in 66 vignettes of 30 to 50 words, representing 15 to 20 items for each concept. Example items from each concept subscale are provided in Table 1. Names used in the vignettes were taken from the lists developed by the National Hurricane Center for naming tropical storms in the Atlantic, with an equal number of male and female names used across the item pool. The reading level of the vignettes was calculated using the Fry Readability Formula and was found to range between a seventh and ninth grade level.

**Table 1:** Example vignettes from each concept subscale.

Concept	Example Item
Bullying	Arlene works as a salesperson for a large company. Her colleagues like to play practical jokes on one another. Arlene is embarrassed by the jokes and has asked her colleagues to stop, but sometimes they still play pranks on her.
Mental disorder	Sara hates getting up in front of people and avoids all types of public speaking. She is dreading having to walk on stage to collect her high school diploma and has asked the school administration whether it can be mailed to her instead.
Prejudice	Dolly is walking in the mall prior to closing. She sees two African American men walking toward her. She doesn’t realize it, but she automatically clutches her purse and walks a bit faster.
Trauma	Danny is fifteen years old. At the end of summer his father was offered a new job and the family moved interstate. Danny is finding it hard to make friends at his new school.

Participants responded to concept subscales in a fixed order: bullying, mental disorder, prejudice, trauma. Within concept subscales, the presentation order of individual items was randomized. After reading each vignette, participants were asked to rate the extent of their agreement that the scenario was an example of the target concept, e.g., “I believe this is an example of bullying”, on a six-point Likert-type scale where 1 = s*trongly disagree* and 6 = s*trongly agree*. Participants then provided demographic data including age, gender, and ethnicity.

### Results and discussion

The full dataset was split into random half subsamples of 175 participants. Mean age and gender breakdown were comparable across subsamples. The scale was developed on Subsample 1, keeping the second subsample quarantined. Within each concept subscale item analysis was conducted, including examinations of variance, corrected item-total correlations, inter-item correlations, and item-response distributions. Items were eliminated if they underperformed on multiple measures. Item analysis and elimination proceeded in an iterative process until each subscale was reduced to 10 items with good internal consistency [13]. Ten items per subscale was selected with the goal of having a measure whose subscales had sufficient reliability to be used singly, while also keeping the full scale at a manageable length. Cronbach’s alpha coefficients and average inter-item correlations for the final subscales in Subsample 1 are provided in Table 2. The final items of the scale are provided in S1 File (a 12-item short form of the Harm Concept Breadth Scale and associated scale statistics are provided in S2 File).

**Table 2:** HCBS Cronbach’s alpha and average inter-item correlations, Study 1.

	Subsample 1		Subsample 2		Full Sample	
	Alpha	AvIC	Alpha	AvIC	Alpha	AvIC
Bullying	.78	.27	.81	.30	.80	.28
Mental disorder	.77	.26	.80	.28	.79	.27
Prejudice	.82	.32	.87	.40	.85	.36
Trauma	.79	.27	.81	.40	.80	.28
Concept breadth	.88	.16	.90	.19	.89	.17
*N*	175		175		350	

The 40 items of the reduced scale were then tested on the second subsample. Cronbach’s alpha coefficients and average inter-item correlations in Subsample 2 were equivalent to, or greater than, those of the development subsample, providing a preliminary indication of the reliability of the final scale. Similarly acceptable reliability was found when testing with the full sample (see Table 2).

Pearson correlations between HCBS subscales ranged from .29 to .47 (all *p*s < .01), providing suggestive evidence of an underlying harm-related breadth factor. While thematic similarities between bullying and prejudice concepts might account for the covariation of these subscales, such an explanation is less intuitive for correlations between the breadth of thematically dissimilar concepts such as bullying and mental disorder.

**Fig 1:**
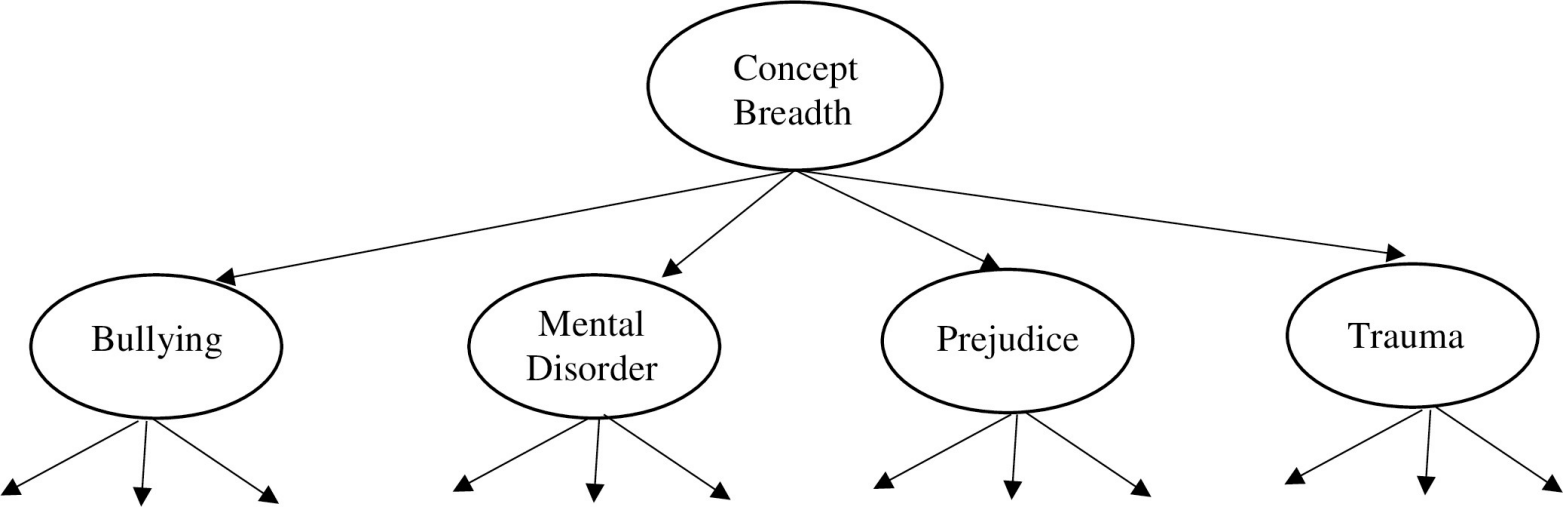
Second-order factor model.

A confirmatory factor analysis where each concept subscale loads on a second order breadth construct was conducted using Mplus version 8 (see Fig 1). Inspection of histograms and Q-Q plots indicated univariate non-normality in a number of items. As these variables would not be expected to be distributed normally in the population, transformations were not performed and the Satorra-Bentler Scaled Statistic was used [14]. Hu, Bentler and Kano [15] found this MLM estimation method to be particularly robust to multivariate non-normality in medium to large samples. For the purposes of the factor analysis, the metric of latent variables was set by fixing the variance of each latent factor to one.

The second order factor model showed acceptable fit using the cutoffs provided by Hu and Bentler [16]: RMSEA = .05 (< .06 = good), CFI = .81, TLI = 0.80 (> .95 = good), SRMR = .06 (< .08 = good). We note that in this and subsequent studies, the specified model fell short of recommended cut-offs for the CFI and TLI fit indices, however optimizing the scale for fit was not our goal, nor is it necessarily plausible given the proposed scale is a deliberately diverse measure of a novel construct. Confirmatory factor analysis was conducted for the primary purpose of demonstrating relationships between the concept subscales and the superordinate breadth construct. Factor loadings of the concept subscales on the superordinate concept breadth factor and descriptive statistics are given in Table 3.

**Table 3:** Descriptive statistics and factor loadings of subscales on concept breadth construct, Study 1.

	Mean (SD)	Factor Loadings		
		Unstandardized	Std. Error	Standardized
Bullying	4.58 (0.74)	1.72	0.35	.86
Mental disorder	3.53 (0.81)	0.73	0.11	.59
Prejudice	4.32 (0.90)	0.83	0.11	.64
Trauma	4.16 (0.73)	0.72	0.13	.58
Concept breadth	4.15 (0.57)			

The findings from Study 1 provide support for the reliability of the HCBS subscales as measures of breadth within concepts. The observed correlations among these subscales are indicative of a generalised harm-related concept breadth construct manifest in systematic individual differences in the tendency to hold expansive versus restrictive definitions of harm-related concepts.

## Study 2: Validation of the HCBS

Study 2 tested the validity of the newly developed HCBS and commenced exploration of its predictors and correlates. Specifically, Study 2 sought to test the predicted link between harm-related concept breadth and harm-based moral judgment and demonstrate its differentiation from established constructs capturing concern for the self and others, and from an inclusive cognitive style.

Moral Foundations Theory [17] proposes there are at least five universal domains of morality upon which all cultures, and individuals within cultures, build their moral codes. The five currently recognised foundations are Harm, Fairness, Loyalty, Authority, and Purity. Two broad groupings have been identified in Western cultures. Loyalty, Authority and Purity are collectively known as the binding foundations as they reflect a belief that the primary goal of moral regulation is to strengthen and protect social relationships such as the family and the nation. Harm and Fairness are individualizing foundations in which the individual is the primary unit of moral concern, and the goal of moral regulation is to protect the individual from harm and injustice [9].

For the purposes of this study, the individualizing foundations were drawn on to capture sensitivity to an individual’s experience of harm and injustice. We anticipated that endorsement of the Harm and Fairness foundations would be positively associated with endorsing broader concepts of prejudice, bullying, trauma and mental disorder, with harm-based morality endorsement most strongly associated with the HCBS. No associations were predicted between concept breadth and the binding foundations.

Justice sensitivity captures concern for injustice directed toward the self and others. The Justice Sensitivity Inventory [18] comprises four subscales reflecting different justice contexts. Victim Sensitivity measures how readily an individual feels unjustly treated and the nature of their reaction to this perceived maltreatment. Witness Sensitivity measures the same dimensions from the perspective of an individual who observes the injustice. Perpetrator and Beneficiary Sensitivity measure perceptions of being the person who carries out or inadvertently benefits from an injustice, respectively. These subscales allow a preliminary exploration of whether people who hold broader concepts of harm are primarily concerned with self- or other-directed injustice. Previous studies using a preliminary measure of harm concept breadth found that both Victim and Witness Sensitivity were significantly positively correlated with holding broader concepts, and we expected to see those results replicated in the present study [12].

Early feedback on this program of research highlighted the need to demonstrate that the HCBS is assessing breadth of specifically harm-related concepts as opposed to a generalized tendency to hold inclusive concepts. We were unaware of any existing measure of generalized category inclusiveness, so sought to develop one for these purposes. Our category inclusiveness index, adapted from McCloskey and Glucksberg’s [19] research on natural categories, assessed people’s tendencies to judge marginal examples of a wide assortment of non-harm-related concepts to be instances of those concepts. We anticipated a positive association between harm-related concept breadth and this new measure of “category inclusiveness”, but that this would not be large enough to indicate redundancy between these two constructs.

In addition to moral foundations, justice sensitivity, and category inclusiveness, we assessed several demographic variables. We predicted a negative association between harm-related concept breadth and age, suggesting that the older the person is, the narrower their concepts. Haslam’s [2] creep theory suggests that concepts of harm have been systematically expanding over the last 50 years. As such, older participants may be expected to have developed their understanding of concepts in a period of time where they were narrower. Further supporting this prediction are claims that expansion in concepts of harm is linked to a heightened degree of vulnerability and fragility in the current college generation, manifesting in such phenomena as safe spaces and trigger warnings [20]. For this reason, we also predicted that higher levels of education would be associated with holding broader concepts.

A particularly strong relationship was expected between political liberalism and harm concept breadth. Early theories of concept creep suggested that expanding the range of people identified as subject to harm and in need of protection aligned with the concerns of a liberal social agenda [2]. This claim is supported by findings from moral psychology that liberal morality is focused almost exclusively on matters of harm and fairness while conservative morality extends into other domains, and that liberals also tend to give greater importance to harm as a moral foundation than do conservatives [21]. These links are also supported by preliminary empirical work [12].

### Method

#### Participants

Participants meeting the eligibility criteria reported in Study 1 were recruited from MTurk. Workers were ineligible if they had participated in Study 1. The sample of 312 comprised 165 men (52.9%), 144 women (46.2%), one participant who identified as queer (0.3%), and one who failed to provide gender information (0.3%).

Participant age ranged from 19 to 74 with a mean of 36.03 years (*SD* = 10.65). 71.4% participants identified as White or Caucasian, 8.6% as African American, 4.7% as Hispanic or Latinx, 8.0% as Asian, and the remaining 4.4% as Native American, mixed race or other ethnicities. Nine participants (3.0%) did not provide a response.

#### Materials and procedure

Participants completed an online survey that included the HCBS, a measure of category inclusiveness, and a number of measures tapping into concern for the self and others.

*Harm Concept Breadth Scale*. The final set of 40 items developed in Study 1 was presented to participants as the Harm Concept Breadth Scale (HCBS). Participants rated the extent to which they believed the scenario was an example of the target concept (i.e., “I believe this is an example of prejudice”) on a six-point Likert-type scale from 1 = *strongly disagree* to 6 = *strongly agree*. These ratings were averaged across items to provide a total harm concept breadth score.

*Moral foundations questionnaire*. The Moral Foundations Questionnaire (MFQ) [9] is a 30-item measure in two parts. The first 15 items measure the relevance of the five moral foundations to an individual’s evaluation of right and wrong. Participants rate considerations such as “whether or not someone suffered emotionally” on a six-point Likert-type scale where 0 = *not at all relevant* and 5 = *extremely relevant*. The second section measures moral judgements of statements pertaining to each foundation e.g. “I am proud of my country’s history”. Responses are again indicated on a 6-point Likert-type scale where 0 = *strongly disagree* and 5 = *strongly agree*. The MFQ yields a score for each moral foundation, with higher scores indicating stronger endorsement of that foundation. Alpha coefficients for the five moral foundations in the present study were: Harm = .75, Fairness = .71, Ingroup = .80, Authority = .79, and Purity = .89.

*Justice sensitivity inventory*. The Justice Sensitivity Inventory (JSI) [18] measures the sensitivity of an individual’s perception of unjust treatment, and their reaction to this treatment. The Inventory comprises four subscales reflecting different contexts: Victim Sensitivity (e.g., “It bothers me when others receive something that ought to be mine”; α = .82), Witness Sensitivity (e.g., “I am upset when someone is treated worse than others”; α = .84), Beneficiary Sensitivity (e.g., “I feel guilty when I am better off than others for no reason”; α = .87) and Perpetrator Sensitivity (e.g. “I cannot stand the feeling of exploiting someone”; α = .85). Ten items in each subscale are rated on a 6-point scale from 0 = *not at all* to 5 = *exactly*.

*Category inclusiveness measure*. The category inclusiveness measure consists of 17 items previously determined to be a marginal exemplar of a given category (e.g. “Is yeast an animal?”, “Is chess a sport?”, “Is a stove a kitchen utensil?”) [19]. Response is a dichotomous “yes” or “no”. Category inclusiveness is assessed as the number of “yes” responses, with higher numbers indicating a greater general tendency to hold broader categories (Kuder-Richardson-20 reliability = .67).

A single item assessed political orientation, “How would you describe your political beliefs” on a 6-point scale from 1 = *extremely conservative* to 6 = *extremely libe*ral. Higher scores are indicative of greater political liberalism. Single item measures of political orientation have been shown to be valid [22]. Participants also provided information relating to age, gender, ethnicity, and educational attainment.

### Results and discussion

The Moral Foundations Questionnaire includes two ‘catch’ items, one which asks the relevance of a person’s math ability to judgments of right and wrong, and another which asks participants whether it is better to do good than to do bad. Participants who provided inappropriate responses (‘somewhat’ to ‘extremely relevant’ for the math question, and ‘slightly’ to ‘strongly disagree’ for the good question) to both questions were excluded. Participant data was retained if only one attention check was answered inappropriately [23]. Eleven participants were excluded on this basis, leaving a final sample of 301 for analysis. Inspection of Mahalanobis distance indicated the presence of multivariate outliers, however Cook’s distances less than 1 across the sample provided evidence that none of the outliers were overly influential and all were retained for analysis [24].

The alpha coefficients and average inter-item correlations for both the subscales and full scale (shown in Table 4) demonstrate internal consistency comparable with Study 1, providing preliminary evidence that the scale is generalizable across samples.

**Table 4:** HCBS reliability statistics and factor loadings of subscales on concept breadth construct, Study 2.

				Factor loadings		
	Mean (SD)	Alpha	AvIC	Unstd.	Std. Error	Std.
Bullying	4.56 (0.77)	.82	.32	1.25	0.19	.78
Mental disorder	3.59 (0.81)	.79	.28	0.66	0.12	.55
Prejudice	4.27 (0.94)	.87	.41	1.11	0.17	.74
Trauma	4.11 (0.72)	.80	.28	0.75	0.11	.60
Concept breadth	4.13 (0.59)	.90	.19			

Unstd. = Unstandardized factor loading, Std. = Standardized factor loading

As with Study 1, positive linear correlations between each of the concept subscales in the range of .32 (Bullying and Mental Disorder) to .50 (Bullying and Prejudice) support the idea of an underlying factor associated with breadth across the four concepts. A second order factor model was estimated in Mplus using the Satorra-Bentler Scaled Statistic to account for multivariate non-normality [15]. In this sample the second order factor model again showed acceptable to good fit based on accepted cutoffs [16]: RMSEA = .06, CFI = .78, TLI = 0.77, SRMR = .07. Factor loadings of the concept subscales on the superordinate concept breadth construct and descriptive statistics are given in Table 4.

Correlations between harm-related concept breadth, moral foundations, justice sensitivity, and category inclusiveness show some of the expected associations but not to the point of redundancy (see Table 5). The hypothesized significant positive correlations were observed between harm-related concept breadth and endorsement of both the Harm and Fairness moral foundations. The lack of association between concept breadth and the three binding moral foundations was also as hypothesized.

**Table 5:** Correlations among all variables, Study 2.

	1	2	3	4	5	6	7	8	9	10	11	12	13	14
1. Concept Breadth														
Moral Foundation														
2. Harm	.44**													
3. Fairness	.38**	.65**												
4. Ingroup	-.02	.14*	-.05											
5. Authority	-.05	.06	-.09	.77**										
6. Purity	-.03	.14*	-.1	.68**	.79**									
Justice Sensitivity														
7. Victim	.03	-.11	-.06	.14*	0.06	.08								
8. Witness	.18**	.11	.01	.12*	0.02	.07	.51**							
9. Beneficiary	.17**	.17**	.09	.22**	.13*	.16**	.28**	.57**						
10. Perpetrator	-.00	-.01	-.18**	.14*	.13*	.12*	.19**	.35**	.39**					
11. Category Inclusiveness	.22**	.05	-.01	.17**	.15*	.15**	.16**	.18**	.17**	.07				
12. Age	-.10	.01	-.01	.01	.03	.02	.02	.08	.07	.01	-.05			
13. Political liberalism	.40**	.36**	.41**	-.20**	-.36**	-.31**	.00	.06	.07	-.09	.05	-.12*		
14. Education	-.02	-.06	-.10	.14*	.11	.09	.08	.02	.06	.05	.09	-.02	.00	
Mean	4.13	3.72	3.71	2.34	2.65	2.45	34.83	35.58	33.18	36.49	7.92	36.03	3.90	4.21
SD	0.59	0.84	0.77	1.07	1.06	1.41	7.36	7.02	8.17	6.69	3.21	10.65	1.46	1.27

* *p* < .05

** *p* < .01

Consistent with our predictions, a small positive correlation between harm-related concept breadth and Witness Sensitivity was observed. However, contrary to our predictions and McGrath et al. [12], concept breadth did not correlate significantly with Victim Sensitivity. An unexpected correlation of harm-related concept breadth with Beneficiary Sensitivity may be attributed to this subscale’s concern with benefiting at others’ expense. These findings suggest that concern for others is more strongly associated with harm-related concept breadth than concern for the self.

The category inclusiveness measure showed a modest positive correlation with harm-related concept breadth. The small magnitude of this correlation, and the fact that all correlations between concept breadth and moral foundations and justice sensitivity subscales persisted when category inclusiveness was partialled out (see S1 Table), demonstrates that harm-related concept breadth is not reducible to generalized category inclusiveness.

As hypothesized, a strong positive correlation was found between political liberalism and harm-related concept breadth. Contrary to our prediction, however, no significant correlation was observed between concept breadth and age, suggesting that in this sample younger people did not hold broader concepts of prejudice, bullying, trauma, and mental disorder than their older counterparts. This finding warrants further investigation, ideally outside an MTurk context. It is plausible that cohort effects may be at play within MTurk samples—a 65-year-old digital gig worker may differ in meaningful ways from a senior citizen who is less digitally literate.

A significant effect of gender was found, women (*M* = 4.22, *SD* = 0.05) having significantly broader concepts than men (*M* = 4.04, *SD* = 0.05*)*, *F* (1,297) = 7.96, *p* < .01, η_p_^2^ = .03. Although not predicted, these results are consistent with the emerging picture of harm-related concept breadth as associated with prosocial concern for others. Many constructs reflecting similar prosocial themes also record gender differences. Women endorse the Harm, Fairness, and Purity foundations more than men [9] and small comparable gender effects have also been found in the justice sensitivity subscales [18].

To provide insight into which of these variables of interest predict the most variance in harm-related concept breadth, we regressed concept breadth on those variables for which significant correlations had been observed. Gender was included in the regression as a dummy variable where male = 0 and female = 1. The sample size of 299 in the regression analysis (excluding the two participants who failed to provide gender information or identified as neither male nor female) provided 80% power to detect a small effect (f^2^ < .05) [25].

Table 6 provides regression coefficients for a multiple regression with all predictors entered simultaneously. The overall model fit was R^2^ = .34. Endorsement of the Harm foundation explained the most variance in harm-related concept breadth, followed closely by political liberalism. The finding that the Fairness moral foundation was not associated with concept breadth supports the proposed specificity of harm-based morality as a predictor of harm-related concept breadth. Category inclusiveness and female gender were also significant predictors of HCBS scores. Neither of the justice sensitivity subscales made an independent predictive contribution.

**Table 6:** Simultaneous multiple regression predicting concept breadth, Study 2.

Predictor measures	B (Std. Error)	β	95% CI
Harm endorsement	0.18 (0.05)	0.25**	[0.08, 0.27]
Fairness endorsement	0.10 (0.05)	0.13	[-0.001, 0.20]
Witness Sensitivity	0.01 (0.01)	0.11	[-0.001, 0.02]
Beneficiary Sensitivity	0.002 (0.004)	0.02	[-0.01, 0.01]
Category Inclusiveness	0.03 (0.01)	0.17**	[0.01, 0.05]
Political liberalism	0.10 (0.02)	0.24**	[0.06, 0.14]
Gender	0.12 (0.06)	0.10*	[0.01, 0.23]

* *p* < .05

** *p* < .001

Overall, the findings from Study 2 suggest that variables associated with a typically political liberal concern for the wellbeing of others are implicated in holding broader concepts of prejudice, bullying, trauma, and mental disorder.

## Study 3: Validation of the HCBS

Study 3 continued the program of construct validation of the Harm Concept Breadth Scale using a sample differing in national origin and age profile. In addition to further testing the reliability and generalisability of the HCBS, Study 3 explored the scale’s relationships with established personality, prosocial, and moral constructs.

Study 3 examined relationships between harm-related concept breadth and the Big 5 personality domains [26]. Because the Agreeableness domain includes prosocial traits, we predicted that it would be positively associated with concept breadth. Because the Open-Mindedness domain incorporates cognitive flexibility, we predicted a significant positive association with holding broader concept boundaries. Negative Emotionality encompasses the tendency to experience anxiety and fear in relation to potential harm and has been associated with attributing hostile intentions in ambiguous situations [27], suggesting a positive relationship with harm-related concept breadth. We saw no theoretical basis for relationships between concept breadth and Conscientiousness or Extraversion and therefore made no predictions regarding these domains.

To clarify further the prosocial aspects of harm-related concept breadth, Study 3 examined its possible associations with components of empathy. In its broadest sense, empathy refers to how an individual responds emotionally and psychologically to the experiences of another. Davis [10] proposed that empathy has four distinct aspects; perspective-taking, fantasy, empathic concern, and personal distress. Perspective-taking is a measure of an individual’s readiness and capacity to see things from another person’s perspective. This combination of other-orientation and capacity for cognitive abstraction suggests a significant positive association with harm-related concept breadth. The empathic concern aspect assesses primarily affective other-oriented feelings of sympathy and concern. As such, we predicted a significant positive association between empathic concern and holding broader concepts. Personal distress shows thematic similarities to negative emotionality in its assessment of self-oriented feelings of anxiety in situations where others are harmed or under threat. On this basis we predicted a significant positive association between this factor and concept breadth. We had no a priori prediction for the relationships between harm-related concept breadth and fantasy, which taps tendencies to put oneself in the position of characters in books, movies and other media.

Moral expansiveness captures breadth of moral concern with respect to the range of entities whose treatment is morally relevant [28]. Consistent with our predictions for Big 5 Open-Mindedness and the perspective-taking aspect of empathy, we expected that those with broader conceptions of moral targets would also have broader conceptions of harm. Nevertheless, because the expansiveness of people’s “moral circle” is theoretically quite distinct from the breadth of their concepts of specific harms, we did not expect this association to be strong, supporting the discriminant validity of the HCBS.

As with Study 2, we predicted a positive association between harm-related concept breadth and category inclusiveness. We also expected to replicate the positive association between political liberalism and concept breadth found in Study 2, and the finding that women hold significantly broader harm-related concepts than men. Study 3 does not include analysis of age or education due to the minimal variance in these variables within an undergraduate student sample.

### Method

#### Participants

Participants were 341 undergraduate psychology students participating as part of a research experience program. They comprised 82 men (24.0%), 257 women (75.4%), and two participants who indicated their gender was non-binary (0.6%). Participant age ranged from 17 to 47 (*M* = 19.77 years, SD = 4.22). The sample identified as Asian (51.3%), Anglo-Australian (32.8%), Australian Aboriginal (0.6%), Middle Eastern (1.8%), European (2.3%), mixed race (5.6%), or other (3.5%), and 2.1% failed to respond.

#### Materials and procedure

Participants again responded to the 40 item Harm Concept Breadth Scale and the 17-item category inclusiveness measure before completing measures of moral expansiveness, empathy, and personality. Finally, participants provided demographic information, including political orientation measured on a 6-point Likert-type scale where 1 *= extremely conservative* and 6 = *extremely liberal*.

*Moral expansiveness scale*. The Moral Expansiveness Scale (MES) [28] captures individual differences in the range of entities considered worthy of moral concern and treatment (α = .92). Scores are based on placement of 30 target entitles (e.g., close friend, dolphin, coral reef) within one of four moral boundaries (inner circle = 3, outer circle = 2, fringes = 1, outside the moral boundary = 0). Higher aggregate scores indicate greater moral expansiveness.

*Interpersonal reactivity index*. The 28-item Interpersonal Reactivity Index (IRI) [10] assesses individual differences in four aspects of empathy; Empathic Concern (α = 80), Perspective-taking (α = 78), Fantasy (α = .79), and Personal Distress (α = .77). For each factor, participants rate 12 items on a 5-point Likert-type scale where 0 = *does not describe me well* and 4 = *describes me very well*.

*Category inclusiveness measure*. Information regarding this measure is as reported for Study 2 (KR-20 = .64).

*The Big Five Inventory 2*. The Big Five Inventory-2 (BFI-2) [26] is a measure of the Big 5 personality factors. It measures each of five domains (Extraversion, α = .85; Agreeableness, α = .77; Open-Mindedness, α = .84; Negative Emotionality, α = .89; Conscientiousness, α = .86) with 12 items, for a scale total of 60 items. Participants rate their agreement that a particular statement describes them on a 5-point Likert-type scale where 1 = *disagree strongly* and 5 = *agree strongly*.

### Results and discussion

Two participants failed to complete the full survey and their data was excluded, leaving a sample of 339 for analysis. The alpha coefficients and average inter-item correlations for the subscales and full scale (shown in Table 7) demonstrate internal consistency comparable with Studies 1 and 2. Positive linear correlations between each of the concept subscales in the range of .23 (Bullying and Mental Disorder) to .47 (Bullying and Prejudice) support the view that harm-related concept breadth is a generalized phenomenon. Confirmatory factor analysis using the Satorra-Bentler Scaled Statistic in Mplus version 8 again indicates good fit for a second order factor model where subscales load on a superordinate breadth factor: RMSEA = .05, CFI = .77, TLI = 0.75, and SRMR = .07 [16]. Factor loadings and descriptive statistics are provided in Table 7.

**Table 7:** HCBS reliability statistics and factor loadings of subscales on concept breadth construct, Study 3.

				Factor loadings		
	Mean (SD)	Alpha	AvIC	Unstd.	Std. Error	Std.
Bullying	4.50 (0.66)	.83	.34	1.11	0.16	.74
Mental disorder	3.72 (0.66)	.73	.21	0.52	0.11	.46
Prejudice	4.53 (0.67)	.78	.27	1.10	0.21	.74
Trauma	4.12 (0.68)	.79	.28	0.73	0.12	.59
Concept breadth	4.24 (0.48)	.87	.14			

Unstd. = Unstandardized factor loading, Std. = Standardized factor loading

Table 8 demonstrates support for the predicted positive associations between harm-related concept breadth and both Agreeableness and Negative Emotionality, although these correlations were only small to medium in size. Contrary to predictions, there was no association between Open-Mindedness and concept breadth, suggesting that intellectual openness or cognitive flexibility do not play a role in harm inflation.

**Table 8:** Correlations between all variables, Study 3.

	1	2	3	4	5	6	7	8	9	10	11	12	13
1. Concept breadth													
2. Moral expansiveness	.10												
Empathy													
3. Personal distress	.10	-.03											
4. Fantasy	.16**	.14**	.21**										
5. Empathic concern	.21**	.17**	.21**	.48**									
6. Perspective-taking	.01	.08	.04	.19**	.46**								
7. Category Inclusiveness	-.12*	.09	-.04	-.03	.03	.05							
Personality													
8. Extraversion	.03	.10	-.30**	.06	.17**	.06	.04						
9. Open-mindedness	.10	.17**	-.14*	.38**	.40**	.37**	-.02	.31**					
10. Conscientiousness	.03	.03	-.26**	-.06	.24**	.18**	-.06	.23**	.16**				
11. Agreeableness	.12*	.07	-.01	.21**	.63**	.47**	.03	.13*	.33**	.35**			
12. Negative Emotionality	.20**	.02	.58**	.29**	.13*	-.13*	-.11*	-.28**	-.04	-.32**	-.16**		
13. Political liberalism	.16**	.17**	.05	0.10	.15**	.00	-.01	.00	.18**	-.03	.09	.08	
Mean	4.24	44.67	14.08	18.02	19.43	18.23	7.89	37.40	43.60	38.53	42.82	37.74	4.20
Standard deviation	0.48	12.84	4.70	4.93	4.45	4.04	3.04	8.27	7.56	8.07	6.60	9.02	0.10

* *p* < .05

** *p* < .01

As predicted, Empathic Concern showed a significant but moderate positive association with concept breadth. However, Perspective-taking had no association, suggesting that it is specifically the affective rather than cognitive aspects of empathy that are most relevant to harm-related concept breadth. Personal distress was unrelated to concept breadth, while Fantasy showed a small but significant positive association. Moral expansiveness did not show any significant association with the range of phenomena identified as bullying, prejudice, trauma or mental disorder. In strong contrast with Study 2, category inclusiveness showed a small negative correlation with concept breadth. This counter-intuitive finding adds further evidence to the conclusion that the breadth of harm-based social concepts can be distinguished from the inclusiveness of other types of categories. As in Study 2, political liberalism was positively associated with concept breadth, although the correlation was small. Results for Study 3 also replicated the finding that women (*M* = 4.31, *SD* = 0.47) have significantly broader harm-related concepts than men (*M* = 4.04, *SD* = .45), *F* (1,335) = 19.96, *p* < .001, η_p_^2^ = .06.

A multiple regression analysis simultaneously regressed harm-related concept breadth on variables showing significant bivariate associations with it. Gender was included in the regression as a dummy variable where male = 0 and female = 1. Sensitivity analysis determined the present study had 80% power to detect a small effect (f^2^ < .05) [25]. Overall model fit was R^2^ = .12 and regression coefficients are shown in Table 9. Negative Emotionality and being female were significant positive predictors of concept breadth, while category inclusiveness was a significant negative predictor. Neither political liberalism, Agreeableness, nor the empathy variables were significant independent predictors. Given intercorrelations among these variables it is likely that they were competing to account for the variance in concept breadth attributable to prosocial concern for others.

**Table 9:** Simultaneous multiple regression predicting harm concept breadth, Study 3.

Predictor measures	B (Std. Error)	β	95% CI
Fantasy	0.04 (0.24)	0.01	[-0.44, 0.50]
Empathic Concern	0.52 (0.33)	0.12	[-0.12, 1.17]
Category Inclusiveness	-0.70 (0.33)	-0.11*	[-1.35, -0.06]
Agreeableness	0.05 (0.21)	0.02	[-0.35, 0.46]
Negative Emotionality	0.26 (0.12)	0.12*	[0.02, 0.50]
Political liberalism	1.76 (1.01)	0.09	[-0.22, 3.74]
Gender	6.79 (2.50)	0.15*	[1.88, 11.69]

* *p* < .05

** *p* < .001

Findings from this study support the potential role of gender in the expansiveness of harm-based concepts. Variables associated with concern for the wellbeing of others continue to be implicated in concept breadth, but results from this study suggest this is better explained by affective than by cognitive factors. The prominence of affective elements in concept expansion is also suggested by positive relationships between harm-related concept breadth and Negative Emotionality.

## General discussion

The current research demonstrates that harm-related concept breadth is a construct related to, but distinct from, sensitivity to harm, moral foundation endorsement, political liberalism, and a general tendency to hold inclusive concepts. It also provides empirical support for the validity and reliability of the HCBS as measure of individual differences in the breadth of people’s concepts of harm.

Findings across three studies demonstrate that harm-related concept breadth is generalized across the disparate concepts of prejudice, trauma, mental disorder, and bullying. It is noteworthy that similar patterns of breadth are found among concepts deriving from different domains of psychology (i.e., social, developmental, and clinical). Individual differences in the breadth of these concepts are correlated with one another despite the disparate nature and sociomoral structure of those concepts. Prejudice and bullying have a clear dyadic structure requiring both victim and perpetrator, whereas trauma and mental disorder arguably involve a victim but do not necessarily involve an identifiable perpetrator. The thematic link among these concepts is their focus on harm, a position that is consistent with the strength of the association between harm-based morality and the HCBS, and that supports Haslam’s [2] assertion that concept creep is specific to harm-based psychological concepts.

The present research also reinforces and extends emerging understandings of the predictors of harm-related concept breadth. This construct appears to be primarily associated with a prosocial concern for others, supporting the first of the two narratives of concept creep presented in the Introduction. Across the two validation studies, prosocial variables such as endorsement of the Harm and Fairness moral foundations (Study 2), sensitivity to witnessing and benefitting from injustice (Study 2), Big 5 Agreeableness (Study 3), Empathic Concern (Study 3), and political liberalism (Studies 2 & 3) were all positively associated with holding broader understandings of bullying, trauma, mental disorder, and prejudice. The replicated finding in Studies 2 and 3 that women held broader concepts of harm is also consistent with this pattern of results, given the established gender differences on many of these prosocial and affective measures [9, 18, 26].

The concern for others reflected in concept breadth appears to draw on affective rather than cognitive appraisals of harm. In the present research, variables associated with cognitive flexibility or abstract concern for others (Big 5 Open-Mindedness, Perspective-taking) were consistently unrelated to concept breadth, whereas variables with an affective component (Empathic Concern, Personal Distress) are consistently related, albeit to varying degrees of strength.

The lack of association between harm-related concept breadth and Victim Sensitivity in the present research provides an early indication that prosocial concern for others may be a stronger driver of concept breadth than concern for the self. This, coupled with the inconsistent or non-existent relationships between holding broader concepts and age and educational attainment, provides a challenge to emerging theories of concept creep that place it in the domain of college campus “snowflakes” and fragile millennials [20, 29]. However, the Study 3 finding that people holding broader concepts of harm tend to be more prone to experience negative emotions suggests that a link between concept breadth and vulnerability does exist.

Although caution is advised when applying cross-sectional findings to longitudinal phenomena, empirical findings concerning individual differences in concept breadth may have implications for theorizing about historical concept creep. Haslam [2] proposed that creep is driven by an ever-increasing sensitivity to harm, reflecting a liberal moral agenda. Our cross-sectional findings that individual breadth is predicted by political liberalism and endorsement of Harm as a moral foundation are consistent with this account of concept creep. Accounts of historical concept creep also imply that older members of society will hold narrower concepts of harm than their younger counterparts. Our findings suggest that if such a cohort effect exists it may not be as strong as other associations with concept breadth. It may be that an individual’s concepts change over the life span as cultural norms shift.

Preliminary findings of a gender difference in concept breadth may also be related to this typically liberal concern for others. Higher concept breadth among women, however, also suggests intriguing theories for the expansion of harm-based concepts at the cultural-historical level. For example, the increasing prominence of women’s voices and experiences in the public sphere over the last 50 years may have contributed to the expansion of harm-related concepts.

The three studies reported here support the notion of harm concept breadth as a generalized construct that captures variation in understandings of a range of harm-based concepts. It is likely, however, that specificities exist between concepts. For this reason, the HCBS was intentionally developed with stand-alone subscales to enable exploration of specific relationships with concepts of interest.

With a fit-for-purpose measure of harm-related concept breadth in place it becomes possible to explore the consequences of concept breadth and concept creep. Haslam [2] suggests that concept creep may have negative outcomes via moral typecasting [30]. When more social phenomena are considered examples of bullying, trauma, prejudice, or mental disorder, more people are identified as victims. Moral typecasting theory suggests that those identified as victims, or moral patients, are perceived to have a commensurate lack of moral agency. Such a loss of agency has been associated with poorer outcomes in mental health contexts and diminished confidence in one’s ability to overcome adversity [31, 32]. The HCBS provides a psychometrically valid and reliable tool for investigating hypotheses such as these.

Future research might also employ the HCBS to predict individual differences in phenomena associated with judgments of harm. For example, the scale might be used to predict judgments of the acceptability of particular social behavior, responses to political messages, or beliefs about the likely impacts of adverse events. It will, however, be important for future researchers to demonstrate whether the HCBS has incremental validity in predicting phenomena such as these, over and above more established constructs such as political ideology, empathy, and broad personality traits. There is early encouraging evidence that a scale of this sort might demonstrate predictive validity over and above such measures. Chan and Haslam [33] found that a preliminary measure of sexism concept breadth, constructed in a similar manner to the HCBS, predicted judgments of moral concern for a female victim of sexual harassment, and of moral condemnation of the male perpetrators, more strongly than either liberalism or gender. The potential utility of the HCBS is not confined to the prediction of relevant behaviors. The scale may also be suitable for use as an outcome variable in research examining factors that may influence understandings of prejudice, trauma, bullying, and mental disorder.

This research presents a number of nonintuitive findings that warrant further investigation. Replication of the age and education null findings with a representative community sample would be particularly valuable given the centrality of these variables to popular framings of concept creep in reports on campus politics and generational differences. We also note the preponderance of Asian-identifying and female students in the Study 3 sample. It is plausible that cultural differences played a role in the significant negative correlation in this sample between harm-related concept breadth and category inclusiveness. A further limitation of this set of studies is the use of a novel measure to distinguish between concept breadth specifically related to harm and a general tendency to have broader category boundaries. Although we believe it represents an acceptable preliminary measure, further research utilising alternate measures of category inclusiveness would be beneficial.

## Conclusion

The three studies reported in this paper indicate that the HCBS is a valid and reliable measure of a potentially important new individual difference variable. Individual differences in the extent to which individuals hold broad or narrow understandings of harm-related concepts such as bullying, trauma, prejudice and mental disorder are related to but meaningfully distinct from constructs involving concern for others and personal vulnerability. We anticipate that the HCBS will serve as a useful tool for explorations of the antecedents and consequences of concept breadth and their implications for theories of cultural concept creep.

## Supporting information

S1 File Harm Concept Breadth Scale. (PDF)

S2 File Brief Harm Concept Breadth Scale. (PDF)

S1 Table Partial correlations controlling for category inclusiveness. (PDF)
