# Supplementary material for: Development and validation of the Harm Concept Breadth Scale: Assessing individual differences in harm inflation
Source: PLoS One. 2020 Aug 18;15(8):e0237732. doi: 10.1371/journal.pone.0237732 (PMC7437461; doi:10.1371/journal.pone.0237732)
Supplement: S1 File — (PDF) [file pone.0237732.s001.pdf]

HARM CONCEPT BREADTH SCALE

Following are descriptions of scenarios that could possibly be examples of various social concepts. You will be asked to read each description and rate whether the description matches your definition of each concept. Please base your response on how ***you*** *define or think about each concept. There are no right or wrong answers, and we are interested in your personal views.*

Bullying

The following descriptions may or may not be considered examples of bullying.
Based on the information you are given, please rate whether you agree that the description is an example of bullying on a scale where:
1 = Strongly disagree, 2 = Moderately disagree, 3 = Slightly disagree, 4 = Slightly agree, 5 = Moderately agree, 6 = Strongly agree

1. When Richard made the basketball team an older boy who failed to make the team punched Richard in the stomach and told him "I'm going to get you". Since then the older boy hasn't spoken to or come near Richard.
2. Last week at school Tammy accidentally bumped into an older girl in the corridor. The girl got angry and called Tammy an idiot loser, before pushing her to the floor and walking off.
3. Katia's friends often laugh at the things she says and tell her what she should do to improve her hair and clothes. Katia sometimes feels upset, but she is grateful to be included in their group.
4. One night, Ed receives a Facebook notification that a boy from school has posted a photo of a person with a severe facial deformity and the words "ha-ha, look at Ed, what an ugly loser". Fourteen other people from school have already commented on the picture.
5. At sport practice, Fiona fell over in the mud. Her knees were scraped and she was crying. A girl from school recorded the fall and posted the video on YouTube. Within two weeks over a thousand people had seen it.
6. Gabi and Hannah hang out together at school. One day, Hannah sends a text message to six of their friends telling them that Gabi kissed one of their boyfriends at a party on the weekend, even though it wasn't true.
7. Joe plays computer games online. One evening, while playing his favorite game, Joe receives messages from a group of anonymous gamers criticizing his gaming ability and telling him to quit the game. Joe feels intimidated and embarrassed and stops playing that game.
8. Kyle has a blog where he shares his thoughts and poetry. Over a few days he receives a lot of anonymous comments making fun of him. Kyle is upset but turns off comments on the blog and continues to write.
9. Bret works as a senior manager in a high-pressure job with tight deadlines. His supervisor often becomes angry and yells at Bret. He also criticizes Bret's performance during team meetings. One time the supervisor was so angry he slammed his fist down on the desk.
10. Arlene works as a salesperson for a large company. Her colleagues like to play practical jokes on one another. Arlene is embarrassed by the jokes and has asked her colleagues to stop, but sometimes they still play pranks on her.

Mental disorder

The following descriptions may or may not be considered examples of a mental disorder. Based on the information you are given, please rate whether you agree that the description is an example of mental disorder on a scale where:

1 = Strongly disagree, 2 = Moderately disagree, 3 = Slightly disagree, 4 = Slightly agree, 5 = Moderately agree, 6 = Strongly agree.

1. Sara hates getting up in front of people and avoids all types of public speaking. She is dreading having to walk on stage to collect her high school diploma and has asked the school administration whether it can be mailed to her instead.
2. Every couple of weeks Laura goes out to a party or a bar and drinks a lot more than she had planned to. Frequently she has so much to drink that she can't get to work the next day.
3. Adrian's wife died in an accident a few months ago. He finds it hard to imagine the rest of his life without her and feels sad all the time. Sleeping is difficult and he and is having trouble getting anything done at work.
4. For as long as she can remember people close to Sally have told her she is moody. She often feels tired and down in the dumps, and yells at her kids sometimes.
5. Greg is 16 and gets in trouble a lot. He has been suspended from school twice this year for starting fights. He sometimes steals money from his mother's purse and last weekend he shoplifted a bottle of bourbon to take to a party.
6. Eight-year-old Nate can't sit still. He talks a lot and often blurts out answers in class without raising his hand. He finds it very hard to sit on the mat at school without fidgeting or getting up and walking around.
7. A couple of times each year Julia will have a terrible nightmare about members of her family being hurt. She wakes up scared and shaking and has difficulty getting back to sleep.
8. Andrea is a straight-A student and had no trouble getting into her preferred college. Her friends call her a perfectionist because she spends hours getting her assignments just right. She often stays up really late planning how to get everything done so she doesn't let anyone down.
9. Bill always feels extremely tense and anxious. He can't relax and has trouble sleeping. Even though he performs well at work, Bill feels miserable and worries constantly about what people think of him.
10. Ever since her divorce two years ago Helen has felt sad, discouraged, and lonely. Going walking used to make her feel better, but now nothing seems to cheer her up and she cries nearly all the time. Six months ago, she quit college because she couldn't concentrate in class.

Prejudice

The following descriptions may or may not be considered examples of prejudice. Based on the information you are given, please rate whether you agree that the description is an example of prejudice on a scale where:

1 = Strongly disagree, 2 = Moderately disagree, 3 = Slightly disagree, 4 = Slightly agree, 5 = Moderately agree, 6 = Strongly agree

1. Josie's seven-year-old daughter is friends with a little girl from a Muslim family. Last week they invited her daughter over after school. Josie felt uncomfortable about her

daughter being in a Muslim home but didn't want to seem rude, so she said they already had plans.

2. Larry doesn't see why racial minorities should get special treatment when it comes to things like job quotas, college entrance and scholarships. He thinks it's unfair and tells his friends it's just reverse racism.
3. Valerie is used to the Asian people who live in her neighborhood. She likes their food and cultural festivals but doesn't see why their shop signs can't all be in English. She thinks it's especially rude when they speak their own language in public places.
4. Frank and his mates like a good laugh. Sometimes their jokes about Jewish and black people are a bit politically incorrect but he figures it's just a bit of fun and they're not hurting anyone.
5. Dolly is walking in the mall prior to closing. She sees two African American men walking toward her. She doesn't realize it, but she automatically clutches her purse and walks a bit faster.
6. Mindy is having trouble with her statistics course and asks Linh for help with their assignment. Linh has only just joined the class, but she is Asian, and Mindy assumes she is good at math.
7. Gordon believes quotas to increase the number of women in some areas of public life are a waste of time. He thinks women make good nurses and teachers but are just not suited to some other kinds of jobs.
8. Owen thinks that women use their looks to get a guy with money. Even though they say they want a nice guy who will treat them well, Owen thinks they won't give an ordinary guy the time of day.
9. William believes discrimination against women is no longer a problem in Western countries and that men and women are now treated equally. Some women, he thinks, just misinterpret innocent things as being sexist.
10. Nicole doesn't have a problem with people being gay but believes marriage should be between a man and a woman. She has suggested to her gay friends that if they want to get married maybe they could call it something else.

Trauma

The following descriptions may or may not be considered examples of a traumatic event. Based on the information you are given, please rate whether you agree that what happened to the person named in the scenario was traumatic on a scale where:
1 = Strongly disagree, 2 = Moderately disagree, 3 = Slightly disagree, 4 = Slightly agree, 5 = Moderately agree, 6 = Strongly agree

1. Fay's 82-year-old mother had to have heart surgery with a 60% survival rate. Fay was extremely anxious leading up to the surgery and fainted in the hospital lounge while waiting to hear the outcome of the surgery.
2. Jerry's mother called to tell him that his 60-year-old uncle had suffered a stroke and passed away. He had always seemed fit and healthy to Jerry and they had only seen each other on the weekend, so it came as a huge shock.
3. Kate's partner called her from the police station to say he had been mugged at gunpoint. On the way to pick him up, Kate was shaking so much she had to pull the car over for a while.
4. Last month Colin was laid off from his job. When the manager told him, Colin felt like he'd been kicked in the stomach. Since then he has felt depressed and worthless and often gets angry with his wife.

5. Grace is six years old. Last year, her parents separated, and her father moved out. Grace didn’t cope well with the change and started wetting the bed and refusing to go to school.
6. Danny is fifteen years old. At the end of summer his father was offered a new job and the family moved interstate. Danny is finding it hard to make friends at his new school.
7. As PTA President, Vicky made a decision that her friend disagreed with. Since then, the friend pretends not to see Vicky whenever they bump into each other at school. She has also convinced other parents to stop inviting Vicky to community events and social gatherings.
8. Teresa's boss often makes her feel uncomfortable. He sometimes massages her shoulders while she's working, and often compliments her on her clothes and body. Teresa dreads going to work, and even though it is a great job, she is thinking of leaving.
9. As a child protection worker, Erin has worked with many children who have been abused or neglected. After all she’s seen and heard, she can't bring herself to go to church anymore and she no longer wants to bring children of her own into this world.
10. As a counsellor working with refugees, Walter builds close professional relationships with many people who have survived war and torture. He often finds himself thinking of the horrors they describe, and has difficulty sleeping. When he does sleep, Walter is regularly awoken by nightmares.
