# Supplementary material for: Development and validation of the Harm Concept Breadth Scale: Assessing individual differences in harm inflation
Source: PLoS One. 2020 Aug 18;15(8):e0237732. doi: 10.1371/journal.pone.0237732 (PMC7437461; doi:10.1371/journal.pone.0237732)
Supplement: S2 File — (PDF) [file pone.0237732.s002.pdf]

BRIEF HARM CONCEPT BREADTH SCALE

The 12-item Brief Harm Concept Breadth Scale (B-HCBS) presents a practical alternative instrument for researchers not wishing to assess the breadth of specific concepts. Reliability statistics are given in ESM Table 1.

ESM Table 1. Reliability Statistics of the Brief Harm Concept Breadth Scale

	Study 1	Study 2	Study 3	Average
Alpha	.75	.78	.70	.74
AvIC	.21	.23	.16	.20
N	350	301	339	

Note. AvIC = Average inter-item correlation

ITEMS

Following are descriptions of scenarios that could possibly be examples of various social concepts. You will be asked to read each description and rate whether the description matches your definition of each concept. Please base your response on how ***you*** *define or think about each concept. There are no right or wrong answers, and we are interested in your personal views.*

Bullying

Based on the information you are given, please rate whether you agree that the description is an example of bullying on the scale provided.

(1 = Strongly disagree, 2 = Moderately disagree, 3 = Slightly disagree, 4 = Slightly agree, 5 = Moderately agree, 6 = Strongly agree)

1. At sport practice, Fiona fell over in the mud. Her knees were scraped and she was crying. A girl from school recorded the fall and posted the video on YouTube. Within two weeks over a thousand people had seen it.
2. Joe plays computer games online. One evening, while playing his favorite game, Joe receives messages from a group of anonymous gamers criticizing his gaming ability and telling him to quit the game. Joe feels intimidated and embarrassed and stops playing that game.
3. Bret works as a senior manager in a high-pressure job with tight deadlines. His supervisor often becomes angry and yells at Bret. He also criticizes Bret’s performance during team meetings. One time the supervisor was so angry he slammed his fist down on the desk.

Mental disorder

Based on the information you are given, please rate whether you agree that the description is an example of mental disorder on the scale provided.

1. Sara hates getting up in front of people and avoids all types of public speaking. She is dreading having to walk on stage to collect her high school diploma and has asked the school administration whether it can be mailed to her instead.
2. For as long as she can remember people close to Sally have told her she is moody. She often feels tired and down in the dumps, and yells at her kids sometimes.
3. Eight-year-old Nate can't sit still. He talks a lot and often blurts out answers in class without raising his hand. He finds it very hard to sit on the mat at school without fidgeting or getting up and walking around.

Prejudice

Based on the information you are given, please rate whether you agree that the description is an example of prejudice on the scale provided.

1. Larry doesn't see why racial minorities should get special treatment when it comes to things like job quotas, college entrance and scholarships. He thinks it’s unfair and tells his friends it’s just reverse racism.
2. Frank and his mates like a good laugh. Sometimes their jokes about Jewish and black people are a bit politically incorrect but he figures it's just a bit of fun and they're not hurting anyone.
3. William believes discrimination against women is no longer a problem in Western countries and that men and women are now treated equally. Some women, he thinks, just misinterpret innocent things as being sexist.

Trauma

Based on the information you are given, please rate whether you agree that what happened to the person named in the scenario was traumatic on the scale provided.

1. Last month Colin was laid off from his job. When the manager told him, Colin felt like he'd been kicked in the stomach. Since then he has felt depressed and worthless and often gets angry with his wife.
2. Teresa's boss often makes her feel uncomfortable. He sometimes massages her shoulders while she's working, and often compliments her on her clothes and body. Teresa dreads going to work, and even though it is a great job, she is thinking of leaving.
3. relationships with many people who have survived war and torture. He often finds himself thinking of the horrors they describe, and has difficulty sleeping. When he does sleep, Walter is regularly awoken by nightmares.
