# Supplementary material for: Development and validation of the Harm Concept Breadth Scale: Assessing individual differences in harm inflation
Source: PLoS One. 2020 Aug 18;15(8):e0237732. doi: 10.1371/journal.pone.0237732 (PMC7437461; doi:10.1371/journal.pone.0237732)
Supplement: S1 Table — (PDF) [file pone.0237732.s003.pdf]

Table S1. Partial Correlations Among All Variables Controlling for Category Inclusiveness, Study 2

	1	2	3	4	5	6	7	8	9	10	11	12	13
1. Concept Breadth													
Moral Foundation													
2. Harm	.44**												
3. Fairness	.39**	.65**											
4. Ingroup	-.06	.13*	-.05										
5. Authority	-.09	.05	-.10	.76**									
6. Purity	-.07	.13*	-.10	.67**	.78**								
Justice Sensitivity													
7. Victim	-.00	-.12*	-.06	.12*	.05	.08							
8. Witness	.14*	.10	.01	.09	.00	.04	.50**						
9. Beneficiary	.14*	.16**	.09	.17**	.09	.12**	.25**	.53**					
10. Perpetrator	-.02	-.01	-.17**	.10	.10	.10	.16**	.32**	.41**				
11. Age	-.09	.01	-.01	.02	.03	.03	.01	.08	.08	.02			
12. Political liberalism	.40**	.35**	.41**	-.27**	-.38**	-.33**	-.01	.05	.09	-.06	-.11		
13. Education	-.04	-.06	-.10	.13*	.09	.08	.07	.00	.04	.04	-.02	-.01	

* *p* < .05, ** *p* < .01
